# Editorial: Rising stars in hematology: 2022

**DOI:** 10.3389/fmed.2023.1334125

**Published:** 2023-11-22

**Authors:** Eleni Gavriilaki, Marcos de Lima, Paolo Fabrizio Caimi, Mutlu Arat

**Affiliations:** ^1^2^nd^ Propedeutic Department of Internal Medicine, Aristotle University of Thessaloniki, Thessaloniki, Greece; ^2^Department of Medicine, The Ohio State University, Columbus, OH, United States; ^3^Department of Medicine, Case Western Reserve University, Cleveland, OH, United States; ^4^Department of Medicine, Istanbul Florence Nightingale Hospital, Istanbul, Türkiye

**Keywords:** hematology, rising stars, future, #CollectionSeries, review

Recognizing the future leaders of Hematology is fundamental to safeguarding tomorrow's driving force in innovation. Indeed, early career researchers have faced a significant and potential “long” impact from the pandemic (1, 2).

Therefore, this Research Topic aimed to showcase the high-quality work of internationally recognized researchers in the early stages of their careers. We highlighted research by leading scientists of the future across the entire breadth of Hematology, and present advances in theory, experiment and methodology with applications to compelling problems. Contributions to the Research Topic were by invitation only. All Rising Star researchers were suggested by established Editors within the Frontiers board in recognition of their influence on the future directions in their respective fields.

All articles submitted to us for this Research Topic underwent a rigorous peer review process. Ultimately, 11 articles were published.

(i) An interesting case report in hematology was presented by analysis of a heterozygous somatic BLNK mutation associated with T-cell LGL (large granular lymphocyte) leukemia and autoimmune diseases. Fouquet et al. highlighted the link between genotype and the unusual clinical phenotype.

(ii) Application of metagenomic next-generation sequencing (NGS) in the diagnosis of visceral leishmaniasis and its treatment evaluation was presented by a case report. Liang et al. indicated the feasibility of the NGS approach and the evaluation of its therapeutic effect.

(iii) A review article presented an update on emerging treatment strategies in rare anemias. Fattizzo and Motta covered a broad spectrum of novel and exciting treatment options in rare congenital and acquired anemias.

(iv) An original article reported an intelligent detection method for plasmodium based on self-supervised learning and attention mechanism. Fu et al. presented an artificial intelligence method of excellent performance for the diagnosis and potentially automatic detection of malaria in the future.

(v) A review article on immune thrombotic thrombocytopenic purpura (iTTP) provided up-to-date knowledge on long-term outcomes and survivorship. Selvakumar et al. focused on epidemiology and potential mechanisms for adverse long-term sequelae of iTTP, best practices in survivorship care, and presented a research agenda for the future.

(vi) A mini review article discussed activation of APC-EPCR-PAR1 axis in sickle cell disease (SCD). Ramadas and Sparkenbaugh reported novel insights into the activation of PAR1 by APC and thrombin, the APC-EPCR-PAR1 axis, and their potential roles in SCD.

(vii) A review article focused on total marrow irradiation in hematopoietic stem cell transplantation for hematologic malignancies. Kerbauy et al. reviewed literatures on applying these novel techniques in autologous and allogeneic transplantation across different clinical entities.

(viii) A study protocol article presented the phase I/II trial of induced HLA-G+ regulatory T cells in patients undergoing allogeneic hematopoietic cell transplantation from an HLA-matched sibling donor. Lysandrou et al. described the study rationale and design, including patient screening, product manufacturing, infusion, and participant follow-up to data collection, management, and analysis.

(ix) A brief research report article highlighted “waitlist mortality” for myeloma patients with limited access to BCMA therapy. Ahmed et al. reported that many patients who were eligible for ide-cel were not able to secure a timely slot, with high mortality rates as a result.

(x) A mini review article explained the interactions of the fibrinolytic and innate immune systems. Whyte has introduced readers into the world of “thromboinflammation” or “immunothrombosis”.

(xi) A review article delineated promises and challenges of a decentralized CAR T-cell manufacturing model. Shah et al. provided in-depth knowledge of the concept of point-of-care (POC) manufacturing or decentralized *in-house* production.

Considering the multi-thematic character of this Research Topic, our hope is to inspire researchers and physicians to continue their explorations into novel advances in Hematology.

## Author contributions

EG: Writing – original draft. ML: Writing – review & editing. PC: Writing – review & editing. MA: Writing – review & editing.
